# Harnessing Crop Diversity through Genetics, Genomics and Phenomics Approaches

**DOI:** 10.3390/plants12081685

**Published:** 2023-04-17

**Authors:** Pasquale Tripodi, Annalisa Cocozza

**Affiliations:** 1 Research Centre for Vegetable and Ornamental Crops, Council for Agricultural Research and Economics (CREA), 84098 Pontecagnano Faiano, Italy; annalisa.cocozza@crea.gov.it; 2 Department of Agricultural Sciences, University of Naples Federico II, Via Università 100, 80055 Naples, Italy

Developing resilient cultivars ensuring adequate productions will be the agriculture industry’s primary challenge in the coming decades to ensure food security, especially with climate change and a growing global population. Massive efforts were made over the past 50 years to develop solutions for crop management and conservation. This resulted in the creation of germplasm repositories that can preserve and reproduce millions of accessions for thousands of extant plant species both ex situ and in situ. This relatively untapped biodiversity is one of the essential components for hastening the discovery of fresh sources of variation with the potential to raise crop yield and resilience. Several technologies in the field of biology offer the chance to thoroughly examine genetic resources at various depth scales, making the comprehension of the mechanisms regulating the complex basis of traits easier, as well as the discovery of novel alleles for breeding programs.

This Special Issue (SI) was launched with the intention to collect articles focusing on the investigation of the still unexplored crop diversity through advanced genomics and phenomics approaches. The SI consists of 11 original research contributions focusing on diverse crops including cereals (rice, maize, and wheat) [1,2,3,4], legumes (cowpea and snap bean) [5,6,7], cherry [8], rocket salad, [9], onion [10], and vanilla [11]. Next generation sequencing methods were mostly used for the study of genomic diversity, while at the phenotypic level, different morphological and qualitative characters were taken into consideration. Genotyping by Random Amplicon Sequencing-Direct (GRAS-Di) was applied for the construction of linkage maps and QTL (Quantitative Trait Loci) mapping in four recombinant inbred line (RIL) populations developed from a nested association mapping panel of Japanese rice (*Oryza sativa* L.) [1]. GRAS-Di is a modified genotyping by sequencing method relying on the sequencing of random amplicons generated in multiple regions of genome. The methodology allowed to cover over 96% of the estimated rice genome sequence at an average depth of 1×. Researchers demonstrated a higher number of single nucleotide polymorphisms (SNPs) markers detected when compared to GoldenGate assay allowing to cover large gaps occurring with the second genotyping platform. The accuracy of GRAS-di allowed precise detection of QTLs for heading date, a main trait underlying adaptation and expansion of rice to different cultivation areas. The described results seemed to be promising for mapping studies in agricultural crops.

In indica rice (*O. sativa* L. var. indica), screening for drought tolerance was performed in mini-hoop facilities projected to avoid rainfall [2]. A total of 74 rice accessions were evaluated for different morphophysiological traits related to shoots and root pathways by subjecting seedlings to two types of treatments: control (100% moisture) and drought stress (50% moisture). A substantial variability in all shoots, roots, and physiological traits measured was observed. Most genotypes showed low to moderate drought tolerance and only a small percentage showed high drought stress tolerance. The study provides useful indication for genotypes to be used by breeders as parents for developing high-yielding drought-tolerant cultivars as well as for genetic and physiological studies toward the understanding of the mechanisms underlying drought tolerance.

An assessment of zinc and iron concentration in different types of Maize (*Zea mays* L.) under different cultivation conditions was performed, in order to define strategies to address micronutrient deficiencies in grain when it is grown in poor soils [3]. Microelement deficiencies (hidden hunger) can lead to a variety of diseases in people whose diets are characterized by inadequate intakes. Researchers tested seventy-seven Zn-enhanced normal, provitamin A, and quality protein maize hybrids across multi environments with different physicochemical soil properties including Zn and Fe soil concentrations. The dissection of genotype by environment (G × E) interaction highlighted a high genetic variation of the hybrids tested and a high G × E influence on iron concentration compared to zinc, thus suggesting a different breeding and management strategies to adopt for determining micronutrient concentration. The study poses the basis for further breeding programs to develop performing micronutrient-biofortified maize cultivars adapted to the sub-Saharan environment.

A study based on the analysis of genetic diversity and its effects on wheat quality was conducted on ‘Solina’, a native cultivar typical of the Abruzzo region (Italy). [4]. A total 24 different ‘Solina’ wheat accession were analyzed with 45,000 DArTSeq (diversity arrays technology sequencing) SNP markers. The genetic dataset was further implemented in the CYMMIT database containing 26 thousand accessions from over the world. The results showed the presence of two main genetic groups with different geographical origin and distinct qualitative traits, which were found to be genotypically related to the native varieties of Turkey and Middle East. The study confirmed that the wide genetic diversity of Solina wheat are an important step for the protection and conservation of its genetic heritage and also have implications for its agronomic management and final use of the product.

Among Leguminosae phenotyping, a strategy was applied in bean pods, which are important both for nutrition and human health due to the high content of dietary fibers and vitamins as well as for the benefits given by flavonoids and phenols. García-Fernández et al. [5] built a snap beans panel (SBP) of 311 accessions by collecting a wide variety of bean pods collected from European gene banks, research institutes, and seed companies. The SBP was characterized by several pod traits including size and shape, fresh color, color of the main coat of the seed, and plant growth habit, thus allowing to determine a wide range of variation. Following a hierarchical procedure, a subset of lines (Core-SBP) was established representing the maximum phenotypic diversity of the SBP. The Core-SBP established in this study was the first attempt to classify snap bean cultivars based on pod-morphological traits and constitutes a valuable source of traits for breeding programs.

Application molecular markers were reported in two studies aimed to assess genetic diversity of cowpea germplasm (*Vigna unguiculata* L.). This crop has a high level of tolerance to harsh heat and drought conditions, thus playing a key role in generating income for farmers of marginal areas. Gumede et al. [6] investigated 90 cowpea accessions, mostly representing the diversity occurring in different areas of Africa, with ~12,000 markers obtained through DArTSeq. The analysis of population structure highlighted the existence of subpopulations having similar ancestry and/or different degree of gene exchanges due to seed exchange activities among farmer and traders. This was reflected by geographical origin of accessions with clusters including individuals from South Africa, Nigeria, Tanzania, and Kenya. The analysis also highlighted a clustering of types reflecting main phenotyping features (e.g., seed shape and seed coat color), breeding target (e.g., growth habit) or consumer preferences. The study shed light on the presence of unique accessions that can be used to establish new breeding programs for developing novel cowpea varieties for the South African market. Furthermore, the study contributes toward planning the management of local cowpea genetic resources better. A different cowpea panel consisting of 97 accessions retrieved from different region of the Mediterranean basin and Southeast Africa was characterized by using a combination of microsatellites, SNP, and SilicoDArT markers [7]. The triple analysis converged to the same evidence, dividing the collection in two main subpopulations which separated Mozambican accessions from Portuguese one. The over-thirty-eight-thousand SNPs obtained allowed to discriminate a putative accession as an outgroup due to isolation causing low geneflow (‘Lardosa’, a Portuguese landrace). ‘Lardosa’ is a landrace of particular interest for traditional farmers economy with a high adaptation to the Mediterranean climate able to maintain a high water use efficiency under water deficit. By providing new insight into the genetic diversity of the cowpea gene pool, this study confirms the necessity of deep characterization of germplasm collection for establishing effective conservation strategies.

A combination of microsatellites and SNPs was also applied in a collection of 100 genotypes of wild and garden rocket (*Diplotaxis tenuifolia* L., *Eruca sativa* L.) [8]. The study reports the first wild rocket genome assembly consisting of 15.3 Gb of sequences assembled in 177,692 contigs for a total of 424 million nucleotides and the “in silico” development of a hundred simple sequence repeats. The authors dissected the genetic diversity of the rocket collection using five microsatellites and nine SNPs converted in cleaved amplified polymorphic sequence (CAPS), providing an unequivocal molecular characterization able to distinguish accessions of the two species, allowing, furthermore, the identification of cases of synonymy within the *Diplotaxis* gene pool. The study provides valuable tools for the community for the genetic and genomic characterization of rocket salad species and for comparative studies with other Brassicaceae crops.

The application of phenotypic and molecular investigation strategies was also described in fruit trees. Toward the establishment of effective conservation and management strategies, a comprehensive analysis of 1442 sweet cherry accessions held in the German National fruit gene bank was reported by Reim et al. [9]. The analysis with pomological descriptors and microsatellite markers facilitated the detection of subpopulations including cultivars with different geographical origins and to infer any errors related to the documentation accompanying each accession. The study provides a proof of concept of combined approaches to determine any occurring redundancy in the collection (duplicates, synonyms) and/or mislabeling in order to improve efficiency toward the reduction in costs for maintenance. Furthermore, the results allow to determine cultivar authenticity and improve the global gene bank collection by including new germplasm materials.

The genotyping by sequencing (GBS) approach was used to examine the genetic structure of the local variety ‘Cipolla Rossa di Acquaviva’ [10]. Given the increasing value on the market and the enlargement of the cultivation area, the aim of the study was to detect any possible contamination with alien germplasm altering the original genetic structure of this native variety. A collection of 49 individuals representing seven populations of ‘Cipolla Rossa di Acquaviva’ was analyzed, including for comparisons of 10 individuals of the variety ‘Cipolla ramata di Montoro’, which is phenotypically similar but with different quality characteristics and growing cultivation area. A panel of 5,011 SNP markers was used for genetic structure analysis; the results supported the hypothesis of contamination of germoplasm marketed as ‘Cipolla Rossa di Acquaviva’. Indeed, two subgroups were identified: a homogenous cluster composed by four subpopulations clearly distinct from another one consisting of three subpopulations which, on the contrary, did not show significant differences with the ‘Cipolla ramata di Montoro’ race. The results obtained provide useful information for the improvement of breeding activities and for genetic traceability, in order to enhance and preserve the value of typical productions.

Ellestad et al. [11] conducted a study on *Vanilla planifolia*, to investigate its evolutionary mechanisms that shaped the genome of cultivated vanilla in Mexico, its center of origin. The genomic structure of *V. planifolia* was compared with the available reference genome ‘Daphna’, evaluating heterozygosity, ploidy, synteny, and SNP correlation at the genome level. Whole genome sequencing was used to compare genome complexity between 15 accessions from different regions and gene pools. High levels of heterozygosity were found in all accessions except one, which showed a low level. The results supported the hypothesis that cultivated vanilla is the result of hybridization and that multiple domestication events underpin the development of several local varieties. These findings may help improve breeding and conservation efforts aimed at preserving the genetic diversity of this crop.

The Special Issue aims to provide new knowledge the broad scientific community involved in the exploitation of the potentialities of crops. We wish to thank all the authors for their important contribution to the research topic and the staff members at MDPI for their support during the editorial process.

## References

[1] Fekih R., Ishimaru Y., Okada S., Maeda M., Miyagi R., Obana T., Suzuki K., Inamori M., Enoki H., Yamasaki M. (2023). High-Density Linkage Maps from Japanese Rice *japonica* Recombinant Inbred Lines Using Genotyping by Random Amplicon Sequencing-Direct (GRAS-Di). Plants.

[2] Kakar N., Jumaa S.H., Sah S.K., Redoña E.D., Warburton M.L., Reddy K.R. (2022). Genetic Variability Assessment of Tropical Indica Rice (*Oryza sativa* L.) Seedlings for Drought Stress Tolerance. Plants.

[3] Goredema-Matongera N., Ndhlela T., van Biljon A., Kamutando C.N., Cairns J.E., Baudron F., Labuschagne M. (2023). Genetic Variation of Zinc and Iron Concentration in Normal, Provitamin A and Quality Protein Maize under Stress and Non-Stress Conditions. Plants.

[4] De Flaviis R., Tumino G., Terzi V., Morcia C., Santarelli V., Sacchetti G., Mastrocola D. (2022). Exploration of the Genetic Diversity of Solina Wheat and Its Implication for Grain Quality. Plants.

[5] García-Fernández C., Jurado M., Campa A., Brezeanu C., Geffroy V., Bitocchi E., Papa R., Ferreira J.J. (2022). A Core Set of Snap Bean Genotypes Established by Phenotyping a Large Panel Collected in Europe. Plants.

[6] Gumede M.T., Gerrano A.S., Amelework A.B., Modi A.T. (2022). Analysis of Genetic Diversity and Population Structure of Cowpea (*Vigna unguiculata* (L.) Walp) Genotypes Using Single Nucleotide Polymorphism Markers. Plants.

[7] Guimarães J.B., Nunes C., Pereira G., Gomes A., Nhantumbo N., Cabrita P., Matos J., Simões F., Veloso M.M. (2023). Genetic Diversity and Population Structure of Cowpea (*Vigna unguiculata* (L.) Walp.) Landraces from Portugal and Mozambique. Plants.

[8] Reis J.M., Pereira R.J., Coelho P.S., Leitão J.M. (2022). Assessment of Wild Rocket (*Diplotaxis tenuifolia* (L.) DC.) Germplasm Accessions by NGS Identified SSR and SNP Markers. Plants.

[9] Reim S., Schiffler J., Braun-Lüllemann A., Schuster M., Flachowsky H., Höfer M. (2023). Genetic and Pomological Determination of the Trueness-to-Type of Sweet Cherry Cultivars in the German National Fruit Genebank. Plants.

[10] Delvento C., Pavan S., Miazzi M.M., Marcotrigiano A.R., Ricciardi F., Ricciardi L., Lotti C. (2022). Genotyping-by-Sequencing Defines Genetic Structure within the “Acquaviva” Red Onion Landrace. Plants.

[11] Ellestad P., Pérez-Farrera M.A., Buerki S. (2022). Genomic Insights into Cultivated Mexican *Vanilla planifolia* Reveal High Levels of Heterozygosity Stemming from Hybridization. Plants.

